# Decoding the energetic trade-offs in green turtle reproduction

**DOI:** 10.1093/conphys/coaf050

**Published:** 2025-07-23

**Authors:** Snehanjana Chatterjee

**Affiliations:** Department of Biological Sciences, Texas Tech University, 2500 Broadway, Lubbock, TX 79409, USA

Understanding the reproductive cycle of endangered green turtles (*Chelonia mydas*) is critical for their conservation, as reproduction strongly influences how populations change over time. Reproduction is hard work. Turtles have to recover between nesting seasons by building up fat at feeding grounds, sometimes taking years to do so. Renato Saragoça Bruno and colleagues (Bruno *et al*., 2025) found that green turtles have two distinct groups of developing eggs in their ovaries, each at different stages of development. The process of yolk formation in turtles is called vitellogenesis. Vitellogenesis starts in the liver, where a precursor protein, vitellogenin, is secreted. This protein helps feed the growing embryos by transferring nutrients into the yolk and making the egg follicles larger. While it was previously thought that yolk development was complete before turtles begin nesting, the research revealed that vitellogenesis continues into nesting season, as indicated by changes in the size of yolk-filled follicles, their nutrient composition and energy consumed during the season. Unlike some turtle species that finish egg development before migrating, green turtles continue developing eggs during nesting (Fig. 1).

**Figure 1:**
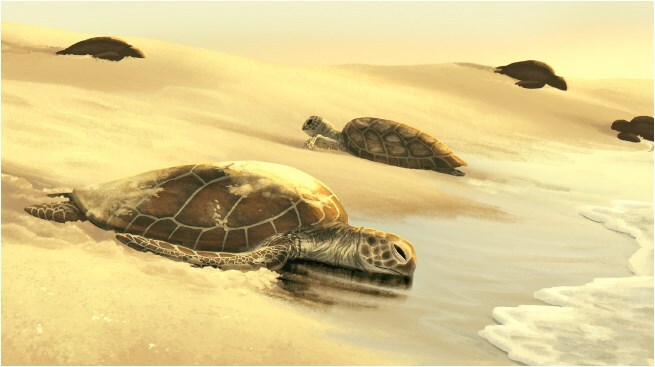
Illustration: Kaitlin Barham (kaitlin.barham@student.uq.edu.au)

To understand how follicle development happens during nesting, the ovaries of 69 green turtles that had been preyed upon by jaguars during three nesting seasons at Tortuguero, Costa Rica, were examined. The group conducted necropsies on the predated green turtles to collect ovaries and oviducts, following remote monitoring of the predation site for jaguar activity. The researchers identified two main follicle groups—dominant follicles, which are destined for ovulation, and non-dominant follicles, which will eventually break down and be reabsorbed by the body.

The smaller dominant follicles grew two-thirds in size as the turtles nested, with the addition of dry matter being the reason for the increase in diameter. The small follicles also contained more water than the large dominant ones. While both small and large dominant follicles showed similar lipid, nitrogen and phosphorus composition, the turtle’s body resorbed phosphorus during follicle breakdown, which was used for eggshell formation. Turtles hydrate by drinking seawater and removing the extra salt through their tear glands and kidneys. But phosphorus is much harder to replace and mostly comes from the turtle’s bones. By quickly pulling phosphorus from follicles that are resorbed, turtles may reduce their bone breakdown during eggshell formation.

Delaying development of all follicles to during the nesting season reduces the weight that turtles must carry by 10 kg. This study showed that even if each turtle used all the energy from resorbing smaller, non-dominant follicles to help grow the larger ones, 83% of them would still not have enough energy to finish the nesting season. To meet their needs, turtles would have to burn fat reserves, break down some developing eggs, or find extra food. But because food available near the nesting beaches is lower in quality and quantity than at their usual feeding grounds, it’s more likely the turtles would have to rely on fat stores or resorb eggs rather than eat more.

Understanding the energetic trade-offs of green turtle reproduction during nesting is vital. As conservation efforts intensify in the face of climate change and habitat loss, recognizing how demanding reproduction is for green turtles is vital to supporting the survival of their populations.

## Notes

**Editor:** Ashleigh Donaldson
